# Thoracomyoplasty in the Treatment of Empyema: Current Indications, Basic Principles, and Results

**DOI:** 10.1155/2012/418514

**Published:** 2012-05-14

**Authors:** Petre Vlah-Horea Botianu, Alexandru Mihail Botianu

**Affiliations:** Surgical Clinic 4, University of Medicine and Pharmacy of Târgu Mures, Gh. Marinescu 66/1, Târgu Mures, Romania

## Abstract

Empyema remains a challenge for modern medicine. Cases not amenable to lung decortication are particularly difficult to treat, requiring prolonged hospitalizations and mutilating procedures. This paper presents the current role of thoracomyoplasty procedures, which allow complete and definitive obliteration of the infected pleural space by a combination of thoracoplasty and the use of neighbourhood muscle flaps (latissimus dorsi, serratus anterior, pectoralis, rectus abdominis, omentum, etc). Recent publications show an overall rate of success of 90%, with a quick and definitive healing. Although rarely indicated in our days, this kind of procedures remain in the armamentarium of modern thoracic surgery. The importance of thoracomyoplasty derives from the fact that it may be a simple and definitive solution for complicated cases of chronic empyema not amenable to standard decortication.

## 1. Introduction

Despite obvious recent advances, empyema remains a challenge for modern medicine. It is a common disease in chest medicine involving important costs and resources, the overall mortality is still high, and the best treatment is still to be defined [1–3]. Surgery is particularly very reluctant to the principles of evidenced-based medicine and very much of the current practice is based on “local protocols” and “personal experience” [4]. The recent recrudescence of tuberculosis (TB) has also brought to attention the pleural complications of this disease [5]. The aim of this paper is to present the current indications, principles, and results of thoracomyoplasty procedures for empyema.

## 2. Historical Background

Empyema is known since Hippocrates, who described the clinical signs and recommended open drainage using cautery [6]. However, lack of knowledge about the pleural physiology made difficult the early attempts of surgical treatment.

Modern management of the empyema starts with the work of the Empyema Commission led by Bell and Graham during World War I. This Commission was created by the US Army to find a solution to the high mortality of patients with parapneumonic empyema. They found that most deaths were the result of the open pneumothorax with lung collapse and respiratory failure that occurred after drainage. They realized the importance of the negative pleural pressure and of the closed drainage and made clear recommendations that are still valid in our days. As a result of the recommendations of the Empyema Commission, there was a dramatic decrease of the mortality of parapneumonic empyema from an average of 30% to 4.3% [7, 8]. 

Thoracoplasty was introduced at the end of the 19th century as a procedure to obliterate empyema cavities by collapsing the chest wall [9]. Many procedures have been described and used, more or less original and more or less popular. The most “radical” was the operation described by Schede [10], which involved resection of the ribs, intercostal spaces, and parietal pleura overlying the empyema; healing of the empyema was achieved by putting in close contact the visceral pleura with healthy tissues represented by the chest wall muscles and subcutaneous fat [10–12].

The operation of thoracoplasty was further developed mainly as a technique to achieve healing of TB by collapsing the lung and had an important contribution to the development of what is now called general thoracic surgery [13]; however, its popularity declined after the introduction of tuberculostatic drugs in the years following immediately the 2nd World War [14]. In our days, thoracoplasty is performed mainly as a solution for chronic empyema and most authors use the technique described by Andrews [15, 16], with or without different modifications [17–19].

Muscle transposition was performed at the beginning of the 20th century by surgeons like Abrashanoff [20], Robinson (1915), Eggers [21], or Archibald [22], but most data published before 1960 were case reports or small series. The technique did not become very popular mainly due to the lack of knowledge about how to mobilize the flaps safely and due to the absence of conditions allowing major thoracic surgery (anesthesia, transfusion, antibiotics, etc.). It became popular in the 1980–90s mainly due to the work of the surgeons (thoracic and plastic-reconstructive) from the Mayo Clinic who showed the value of different muscle flaps in the treatment of severe intrathoracic infections [14, 23, 24].

## 3. Modern Indications

From the very beginning, it must be clearly stated that thoracomyoplasty procedures address to a very small group of patients with empyema. First of all, most patients with parapneumonic effusions and empyema can be cured by antibiotics and thoracocenthesis or tube-thoracostomy, with no need to perform major surgery [25]. If this is required, the first option is lung decortications, which obliterates the space by reexpanding the lung and has several very important advantages: no chest wall mutilation, functional recovery of the collapsed lung, and no significant long-term sequelae [26]. The possibility to perform this procedure using a minimally invasive approach makes it even more attractive by reducing the morbidity and the postoperative pain and by improving the esthetic aspect [27]. Decortication performed through video-assisted thoracic surgery (VATS) is now the first option for most patients with empyema requiring major surgery [28].

However, lung decortication (open or VATS) requires two major conditions in order to be successful. First, there must be a cleavage plane allowing to decorticate the lung; if this plane does not exist or is not clearly defined, the procedure becomes difficult or even impossible due to the bleeding and air leaks that occur during the dissection. Second, the underlying lung parenchyma must have the ability to reexpand and completely obliterate the pleural space. If these two conditions are not fulfilled, lung decortication becomes a hazardous and very risky procedure and thoracomyoplasty becomes an option that should be taken into consideration [29].

Therefore, thoracomyoplasty for empyema is nowadays indicated in the following situations:

- absence of a cleavage plane allowing the surgeon to decorticate the lung,
- inability of the lung to reexpand and completely fill the pleural space,
- postoperative empyema, where decortication is not possible or has failed,
- presence of bronchial fistulae: their safe closure is mandatory and suture-reinforcement using muscle flaps, with or without a thoracoplasty, is a good and safe option,
- presence of unresectable lesions in the lung parenchyma: this diseased space must also be filled with well-vascularized tissue.

TB empyema is not by itself an indication for thoracomyoplasty, although, in the past, different collapse techniques were used to treat TB. However, TB patients with prolonged medical treatments and parenchyma lesions present more frequently with the aforementioned features, making them candidates for thoracomyoplasty. In our experience with this kind of surgery, almost one half of the cases had different forms of TB disease.

Thoracomyoplasty involves opening of the chest, resection of some parts of the chest wall, and dissection of muscle flaps on large areas. Therefore, it is a major procedure and the ability of the patient to tolerate it should be clearly assessed when planning the surgery. The preoperative evaluation should be basically the same as for any major thoracic procedure [30] Due to the esthetic disturbance of the chest, thoracomyoplasty procedures are less attractive for young and female patients.

This kind of surgery involves a certain degree of chest wall mutilation and some functional impairment. As a matter of fact, one of the main objective of the modern techniques is to minimize these adverse effects. However, these aspects must be clearly discussed with the patient before the operation and a written consent should be obtained [31, 32].

## 4. Basic Principles and Techniques

### 4.1. Preoperative Preparation

It is essential in this kind of surgery. Most patients present with an altered biological status secondary to the infection and significant associated diseases requiring a careful reequilibration. Antibiotics should be administered according to the sensitivity of the microorganisms involved. Local control of infection should be achieved by thoracocenthesis, tube-thoracostomy, or even open-window. Daily lavages of the empyema cavity are required to achieve an operative field as clean as possible.

### 4.2. Planning the Procedure

It must be done very carefully and several factors should be clearly assessed:

- location and dimensions of the empyema cavity, which can be well evaluated using modern CT scans with 3D reconstructions;
- presence or absence of bronchial fistula, whose safe closure is mandatory;
- available flaps—previous surgery may damage some of the vascular pedicles, making some flaps impossible to raise—that is, myocardial revascularization using the mammary artery or subcostal laparotomy compromise the ipsilateral rectus abdominis, standard posterolateral thoracotomy sections, the latissimus dorsi, and so forth;
- the morbidity generated by the use of a certain flap and the complexity of the mobilization.

### 4.3. Technical Details

In most cases a posterolateral thoracotomy skin incision is made. After sectioning of the subcutaneous fat, the latissimus dorsi and the serratus anterior are mobilized partially to allow access to the empyema cavity. After entering the empyema cavity, the topography of the lesion is carefully evaluated and the final decision is made. We prefer to start with a complete mobilization of the flaps, according to the topography of the lesions:

- *the latissimus dorsi muscle*:
  - the standard mobilization is based on the thoraco-dorsal vessels, resulting in a large flap that reaches almost any part of the thorax [33]; it is probably the most used flap in both plastic-reconstructive and thoracic surgery,
  - the reversed latissimus dorsi flap is based on the secondary blood supply represented by some perforator branches from the last intercostal and first lumbar vessels: it is a much more difficult flap with a variable anatomy and limited arch of rotation, but it may be a good solution for defects located in the supradiaphragmatic area [34, 35];
- *the serratus anterior *has as main blood supply a branch from the thoraco-dorsal vessels (not recognized by the Nomina Anatomica) which allows the mobilization of the entire muscle. The secondary blood supply represented by the lateral thoracic vessels supports only a limited portion of the muscle. When a full mobilization of the serratus anterior is performed, it results a flap with a volume that is comparable with the latissimus dorsi and can reach any point located in the upper half of the thorax, including the hilar region [35, 36]. Due to the common blood supply, the latissimus dorsi and the serratus anterior may be raised together using the thoraco-dorsal vessels;
- *the pectoralis major* may be raised in more ways:
  - using the thoracoacromial vessels, which results in a flap with good mobility that is useful for defects located in the apex of the chest,
  - using the perforator branches from the internal mammary and the anterior intercostal vessels, which results in a flap with a limited mobility suited for defects located in the upper paramediastinal area [37, 38];
- *the rectus abdominis* flap can be raised using the superior epigastric vessels, which continue the internal mammary artery and vein; although the tip of this flap may reach the base of the neck, it is usually used for defects located in the lower half of the chest [39];
- *the omentum*—although it is not a muscle, it is used with the same purposes and principles; it is mobilized using the left or right gastroepiploic vessels and brought inside the chest through a small diaphragmatic opening. It is an excellent material for closure of large bronchial fistulas [40];
- *other flaps rarely used* to fill an infected pleural space include the trapezius, subscapularis, infraspinatus, external oblique, and teres major. In the available literature, there is no important experience with them and they should be taken into consideration mainly when other more common neighbourhood muscles are not available [41, 42].

Introduction of the flaps inside the chest requires a second opening, which is done by a limited rib resection (10–15 cm length, no more than one rib) to allow safe passage of the flap and its blood supply. The flaps must reach the defect without any tension or torsion. At the end of the procedure, the muscle flaps must remain with a good blood supply, both arterial and venous. As for any procedure that involves muscle flaps, severe ischemia with necrosis will result in complete failure of the operation [32].

Associated thoracoplasty is often necessary to achieve complete obliteration of the infected pleural space. In opposition to many classic procedures based on extensive rib resection, thoracoplasty should be as limited as possible to avoid major chest difformity and long-time sequelae. Rib resection must never expand beyond the edges of the empyema cavity. We also believe that preservation of the first rib is mandatory to avoid shoulder asymmetry and functional disturbances. When mobilized carefully, the muscle flaps may fill most of the empyema cavity, as well as the dead angles and the cul-de-sacs, thus reducing the extent of the rib resection. The resection of the ribs should be made using a subperiosteal plane, thus allowing some regeneration of bone tissue, which improves the long-time rigidity of the chest wall. Creation of intercostal flaps is very easy after thoracoplasty, by simply sectioning the remaining pleuro-periosto-intercostal plane through the bed of the resected ribs.

**Figure 1:**
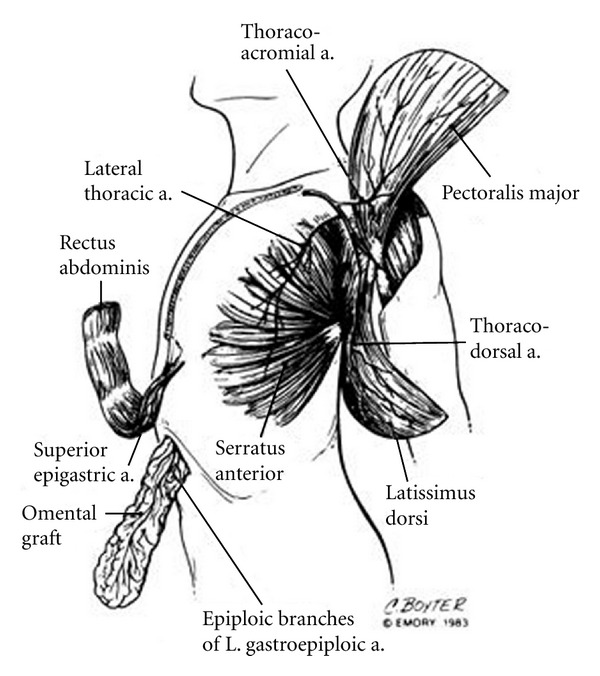
Anatomic drawing of the blood supply of the most used extrathoracic muscle flaps that are transposed inside the chest to obliterate infected spaces. From Miller et al.—Single-stage complete muscle flap closure of the postpneumonectomy space: a new method and possible solution to a disturbing complication, Ann Thorac Surg 1984; 38 : 227-31.

**Figure 2:**
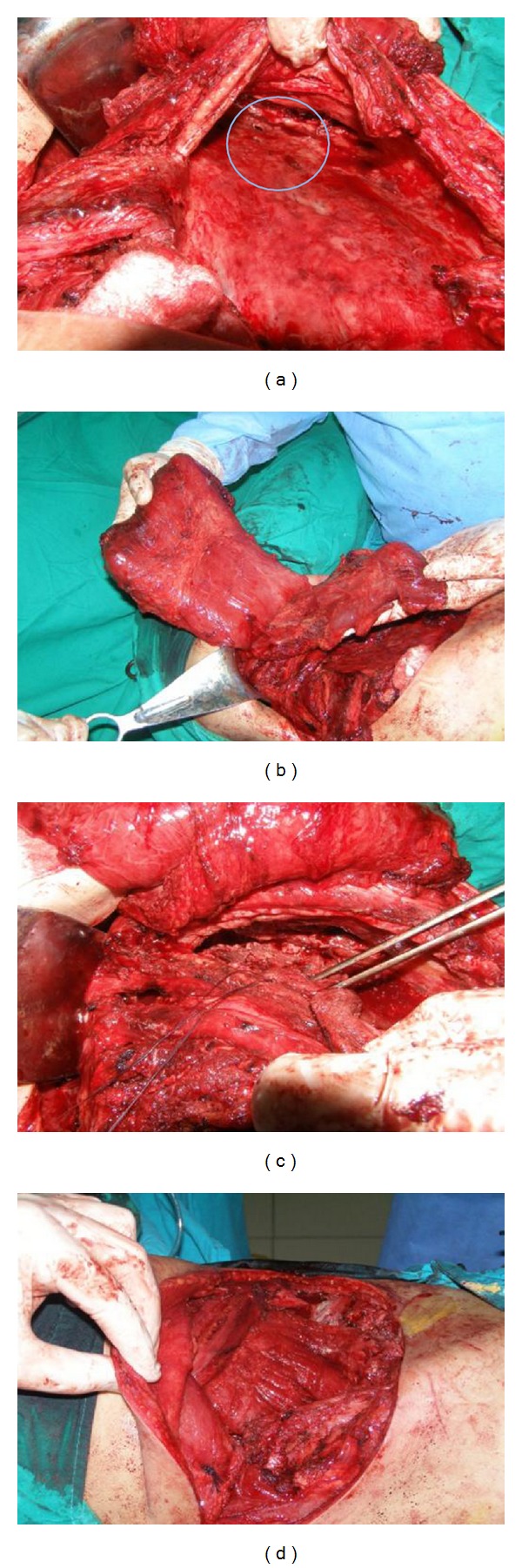
TB empyema with multiple bronchial fistulae solved by thoracomyoplasty— personal collection. (a) Aspect of the cavity with multiple large bronchial fistulae (encircled area). (b) The latissimus dorsi and serratus anterior flaps. (c) Closure-reinforcement of the bronchial fistulae. (d) Final aspect at the end of the procedure. Note the associated rib resection and the complete obliteration of the empyema cavity with the use of the muscle flaps.

At the end of the procedure, the empyema cavity must be completely obliterated by this combination of muscle flaps and thoracoplasty. Especially for big cavities, a certain compromise must be found to avoid both mobilization of multiple muscle flaps and an extensive chest wall resection (Figures 1 and 2).

Drainage of the empyema cavity is mandatory; we usually use an irrigation-aspiration system that allows not only drainage of the cavity, but also postoperative lavages with different antibiotic and disinfectant solutions. If the mobilization of the flaps is an extensive one (as it happens in most cases), the subcutaneous space must also be drained to avoid postoperative seroma. The wound is closed primary with separate stitches [30, 35].

## 5. Personal Experience and Results from the Literature

We started to use thoracomyoplasty with extensive mobilization and intrathoracic transposition of flaps since 2003 and have recently published a detailed analysis of our first 76 cases [35, 43, 44]. This is a group of desperate patients with intrathoracic infections that were not amenable for lung decortication and/or resection. As particular clinical and pathological aspects of our small series, we mention the high proportion of

- active TB cases (36 cases, 47%) with 28 patients still having positive bacteriologic cultures and 7 patients with multi-drug-resistant infections,
- postoperative empyema (13 patients, 17%),
- frank intrapleural rupture of a pulmonary cavity (18 patients, 24%),
- bronchial fistulae (26 patients, 34%).

In our series, we encountered an overall mortality of 5% (4 patients). Local complications included recurrence of the intrathoracic infection in 4 patients (5%) that required a modified open-window procedure, minor skin necrosis solved by simple excision in 3 patients (4%), and external thoracic fistula solved by local lavages in 2 patients (3%). Postoperative hospitalization ranged between 4 and 180 days with an average of  40 ± 5  days (confidence level: 95%). At 3-month followup, 66 patients (91%) of the survivors returned to an almost normal life compared with their preoperative status.

Other authors have recently published their experience with this kind of surgery (with or without different technical details) with quite similar results, showing that in selected cases thoracomyoplasty may be a valuable solution [19, 45–49]. There seems to be a an overall mortality around 5% with a success rate (defined as chest closure and cure of the empyema with no recurrence of the intrathoracic infection) of over 90%.

There are many unsolved problems since due to the rarity of these procedures and the great heterogenicity of the patients we cannot talk about randomized studies or even fair retrospective comparisons. As a direct result, some questions are still to be answered: which is the best flap and when and how a certain flap should be mobilized, what is the number of flaps that should be mobilized, what should be the extent of rib resection, and so forth.

## 6. Conclusions

Thoracomyoplasty remains a valuable surgical solution for difficult empyema cases not amenable to lung decortication. Its value comes from the fact that it may be one of the last solutions for some desperate cases. It achieves healing by immediate complete obliteration of the empyema cavity. Compared to classic thoracoplasty procedures—including the operation described by Andrews—the use of muscle flaps mobilized using techniques borrowed from plastic-reconstructive surgery helps improving the results mainly by limiting the extension of the rib resection and by filling the empyema cavity with a well-vascularised tissue, which is able to fight against infection and promote healing. Although not commonly indicated in our days, thoracic surgeons should be familiar with this kind of procedures [50].
